# Prevalence of 10-Year Risk of Cardiovascular Diseases and Associated Risks in Canadian Adults: The Contribution of Cardiometabolic Risk Assessment Introduction

**DOI:** 10.1155/2013/276564

**Published:** 2013-04-30

**Authors:** Solmaz Setayeshgar, Susan J. Whiting, Hassanali Vatanparast

**Affiliations:** College of Pharmacy and Nutrition, Division of Nutrition and Dietetics, University of Saskatchewan, Saskatoon, SK, Canada

## Abstract

*Background*. Cardiovascular disease (CVD) is the leading cause of death in adult Canadians. Cardiometabolic risk (CMR) derived from 10-year risk of cardiovascular diseases and metabolic syndrome (MetS) needs to be evaluated in Canadian adults. *Objective*. To determine CMR among Canadian adults by sociodemographic and lifestyle characteristics. *Subjects and Methods*. Data from the Canadian Health Measures Survey (CHMS), Cycle 1, 2007–2009, was used. Framingham Risk Score (FRS) was implemented to predict 10-year risk of CVD, and metabolic syndrome was identified using the most recent criteria. The 10-year risk of CVD was multiplied by 1.5 in individuals with MetS to obtain CMR. Data were weighted and bootstrapped to be able to generalize the results nationally. *Results and Conclusion*. CMR gave more accurate estimation of 10-year risk of CVD in Canadian adults from 30 to 74 years than using only FRS. The 10-year risk of CVD in Canadian adults significantly increased when CMR was taken into account from 8.10% to 9.86%. The CVD risk increased by increase in age, decrease in education, and decrease in physical activity and in smokers. Canadians with medium risk of CVD consumed significantly less fruit and vegetable juice compared to Canadians with low risk. No other dietary differences were found.

## 1. Introduction

Cardiovascular diseases (CVDs) are the leading cause of death in Canadian men and women [1]. In 2008, CVD accounted for 29% of all deaths in Canada (28.0% in males and 29.7% in females) [2]. Over $20.9 billion is spent each year in Canada for physician services, hospital costs, lost wages, and decreased productivity related to heart disease and stroke [2].

Population-based strategies in assessing the risk of CVD and cost-effective interventions would play a fundamental role in decreasing rates of morbidity and mortality from CVD. The Framingham Risk Score (FRS), developed through Framingham Heart Study in 1971 to 1974, is the most commonly recommended assessment tool for evaluating 10-year risk of CVD in the United States [3]. The National Cholesterol Education Program (Adult Treatment Panel III) recommends using FRS to assess 10-year risk of CVD [4]. The FRS has been validated for the Canadian population by a committee of clinicians and researchers [3]. However, increasing evidence has indicated that CVD risk algorithms such as FRS may underestimate an individual's actual risk and postpone the initiation of appropriate intervention probably due to not considering waist circumference and triglyceride [5, 6].

Metabolic syndrome (MetS) is a clustering of five chronic disease risk factors, including abdominal obesity, dyslipidemia (elevated triglycerides (TG) and reduced high-density lipoprotein cholesterol (HDL-C) level), hypertension, and elevated fasting plasma glucose (FPG) [7]. MetS is considered to be the main contributor to CVD and diabetes [7, 8]. Regardless of ethnic diversity, the risk of CVD doubles with MetS; also, the risk of diabetes increases fivefold [9–12]. According to our recent data from Canadian Health Measures Survey (CHMS), 2007–2009, 18.31% of Canadians aged from 12 to 79 y had MetS [13]. However, an increase in relative risk of CVD cannot be used to evaluate absolute risk. Furthermore, reported relative risk of CVD and its association with MetS are not comparable between studies as not all studies take into consideration potential confounders.

The new approach on assessing “cardiometabolic risk (CMR)” or “global cardiometabolic risk” considers the factors that go beyond the traditional risk factors [5, 6]. In 2009, the Canadian Cardiometabolic Risk Working Group suggested that CMR represents the comprehensive catalogue of factors related to CVD and Type 2 diabetes [5, 6]. Therefore, in evaluating CMR, both MetS and 10-year risk of CVD are considered. The calculation of FRS followed by the evaluation of the presence or the absence of MetS helps identify individuals whose risk might be underestimated. Therefore, in order to evaluate CMR, both the risk factors of MetS and the risk factors used to calculate 10-year risk of CVD are considered. This novel approach, CMR, has been used to identify the risk of CVD at the individual level [5, 6]; however, to our knowledge no nationally representative study has used this method to evaluate the risks at the population level. Moreover, it is not understood if calculating CMR could possibly change the prevalence of the 10-year risk of CVD at a population level.

Different dietary patterns impact cardiometabolic components in different ways. The Dietary Approaches to Stop Hypertension (DASH) diet or Mediterranean diet are inversely associated with cardiometabolic abnormalities [14–16]. Similarly, coronary heart disease is positively associated with the Western diet as opposed to the prudent diet [17]. Our recent findings from CHMS indicate that Canadians with MetS consumed less dairy products, sugar sweetened beverages, and dietary fat, but had a greater intake of diet soft drinks [13]. The dietary behavior of Canadians at different levels of CVD risk has yet to be explored.

The first objective of the present study was to determine the 10-year risk of CVD in Canadian adults and potential changes at population level when CMR is taken into account. The second objective was to determine 10-year risk of CVD in Canadian adults by different sociodemographic characteristics. Further, we determined the dietary intakes of Canadians in risk categories of 10-year risk of CVD. 

## 2. Subjects and Methods

### 2.1. Study Population

Data from the Canadian Health Measures Survey (CHMS), Cycle 1, 2007–2009, conducted by Statistics Canada in partnership with Health Canada and Public Health Agency of Canada, was used. CHMS is a nationally representative survey collecting health indicators among a sample of approximately 5,500 Canadians aged 6 to 79 y (representative of 96.3% Canadians through multi-stage sampling strategy). The survey consists of two stages: the first stage is self-reported data collection through interviews, and the second stage consists of taking direct physical measurements at Mobile Examination Centers (MEC). Individuals living on reserves or in other aboriginal settlements in the provinces, remote areas, institutional residents, and full-time members of the Canadian Forces were excluded from the survey. The sampling weights, provided by Statistics Canada, were calculated by multiplying the selection weights for collection sites and the selection weights for dwellings (obtained from 2006 Census of Canada), adjusted for nonresponse [18]. The final individual weight was obtained after converting the household weights followed by adjustment for nonresponse at the interview stage and the MEC stage. For the purpose of our study, we excluded individuals who were either under the age of 30 years or over the age of 74 years, as well as nonfasting subjects, pregnant women, and those with confirmed diagnosis of heart disease. The final number of respondents for the current study was 1,293.

### 2.2. Indicators Needed to Estimate 10-Year Risk of CVD

To estimate the 10-year risk of CVD, categorical charts and tables based on FRS were used [19]. Separate score sheets were developed for each sex according to age, blood pressure, total cholesterol (Tchol), low-density lipoprotein cholesterol (LDL-C), and high-density lipoprotein cholesterol (HDL-C) categories. The points assigned for each risk factor are based on the value for the *β*-coefficient of the proportional hazards regressions [19]. Further, we factored the presence or absence of diabetes and smoking status (smoker versus nonsmoker) in our estimations. 

### 2.3. Creating Variables of Interest Using CHMS Dataset

Using either LDL-C or Tchol is optional in assessing CVD risk [3]. LDL-C could be found in only fasted CHMS subsample; whereas, Tchol was available in both fasted and nonfasted subsample. For the current study we used fasted subsample and we evaluated both LDL-C and Tchol. Daily smokers, occasional smokers, or those who stopped smoking less than a year ago were classified as smokers. To recognize individuals with undiagnosed diabetes, Glycated Hemoglobin (HbA1c) ≥ 6.5% was used as the criteria [20]. To obtain the total number of diabetics, cases of self-reported diabetes were added to the individuals with undiagnosed diabetes. Other variables such as age, HDL-C, and systolic and diastolic blood pressure were used to obtain the risk score [19]. All variables were categorized according to the corresponding cutoffs defined in FRS [3]. We reported 10-year risk of CVD using two approaches: (1) 10-year risk of CVD by implementing LDL-C and (2) 10-year risk of CVD by implementing Tchol. Both LDL-C and Tchol cut-points are specified in FRS. 

### 2.4. Calculating CMR

To include the CMR in the estimation of 10-year CVD risk, we first identified individuals with MetS. We applied the most recent unified definition established in 2005 by the International Diabetes Federation (IDF), in collaboration with American Heart Association/National Heart, Lung, and Blood Institute (AHA/NHLBI), for adults. The presence of at least three of the following five metabolic risk factors constitutes a diagnosis of MetS: abdominal obesity (shows the cut-points used in the current study) [21], elevated TG level (1.7 mmol/L), reduced HDL-C level (1.0 mmol/L in males; 1.3 mmol/L in females), elevated blood pressure (BP) (systolic ≥ 130 and/or diastolic ≥ 85 mm Hg), and elevated FPG level (≥5.6 mmol/L). We followed IDF's recommendation by using ethno-specific cutoffs for waist measurement [21]. Individuals who have already been diagnosed as hypertensive, diabetic, or those who were using antihypertensive drugs were also included. In the next step, the 10-year CVD risk among individuals with MetS was multiplied by 1.5 [6]. 

### 2.5. Prevalence of CMR by Different Level of Sociodemographic Characteristics

The age- and sex-specific groups in our analysis were males and females aged 30–34 y, 35–39 y, 40–44 y, 45–49 y, 50–54 y, 55–59 y, 60–64 y, 54–69, and 70–74 y. Four levels of education based on the highest level achieved by any member of the household were defined as less than secondary school graduation, secondary school graduation, and some postsecondary, or postsecondary graduation. Four economic status levels were based on the total household income and the number of individuals in the household. Only two ethnic groups, that is, White and non-White, were created due to few numbers of non-White individuals in various ethnic groups in that category. Physical activity was measured in CHMS using a questionnaire which calculated the total daily leisure time energy expenditure (EE) values (kcal/kg/day) during leisure time activities. Respondents were subsequently categorized into “active” (EE ≥ 3), “moderate” (1.5 ≤ EE < 3), or “inactive” (0 ≤ EE < 1.5) physical activity. 

### 2.6. Dietary Assessment in Three Levels of Risk

 In CHMS, usual dietary intake was collected through a semiquantitative food-frequency questionnaire. Dietary intake was collected based on the frequency of daily, weekly, monthly, or yearly consumption. Food groups in CHMS were defined as follows: meat and fish (e.g., red meat, organs, hotdogs, sausage or bacon, seafood, eggs, beans, nuts), grains, fruit and vegetable (e.g., hot/cold cereal, white bread, brown bread, any kind of rice, any kind of pasta, fruit, and vegetable including potato), milk and dairy product (e.g., milk, cottage cheese, yogurt, and ice cream), dietary fat (regular-fat salad dressing or mayonnaise, regular-fat potato chips, tortilla chips, and corn chips), water and soft drink (e.g., regular soft drink, sport drink, fruit drink, diet soft drink, and fruit/vegetable juice). The questions for each section were only on the frequency of consumption without quantifying the amount of intake. The respondents were asked to state the frequency of consumption per day, week, month, or year. Responses “I don't know” and “Refused to answer” were not included [22]. With the exception of the section on water and soft drink consumption, all other dietary intake questions were included for the first time for CHMS. Water and soft drink consumption questions were derived from the National Population Health Survey, NPHS, Cycle 6 [23]. The verification of questionnaire responses was conducted at the end of completing data collection at each site by reviewing and adjusting using notes recorded by interviewers. The food groups used in this study are consistent with what was used in CHMS. For the purpose of the current study all dietary consumption data was converted into a daily frequency of consumption (times/day).

Based on the FRS scores, we categorized participants in three levels of risk: low, medium, and high [4]. The low number of individuals at high risk level resulted in wide confidence intervals for estimates. Therefore, we only compared individuals at medium risk with individuals at low risk for dietary differences.

### 2.7. Data Analysis

To meet the first objective, the 95% confidence intervals (CI) of 10-year risk of CVD and CMR estimates were compared. No overlap in 95% CIs of the estimates was considered significant statistical difference at 0.05 [24]. For the second objective, we report the difference in 10-year risk of CVD by sex, age, and each sociodemographic characteristic using independent sample *t*-test or one-way ANOVA at sample level. At the population level, data was weighted and bootstrapped and comparisons across groups were performed using 95% CIs overlap. 

To examine possible differences between dietary intakes across three levels of 10-year risk of CVD (Objective 3), the same method of using 95% weighted CIs overlap was implemented. 

Data manipulation, cleaning, and creation of new variables were done using PASW Statistics 19. All statistical analyses were conducted by STATA/SE 11, StataCorp. As per Statistics Canada's recommendation, all analyses were weighted and bootstrapped in order to be representative of the Canadian population. The degrees of freedom of 11 in CHMS Cycle 1 were limited due to sampling structure. Alpha was set at 0.05. 

## 3. Results 

### 3.1. 10-Year Risk of CVD in Canadian Adults

The CHMS participants in our study (*n* = 1,293) represent 17, 250,853 Canadians aged 30–74 y. Overall, the risk of CVD in Canadian adults using LDL-C indicator and Tchol was 8.10% ± 8.89 (CI: 7.33–8.87) and 7.70% ± 8.75 (CI: 7.07–8.32), respectively. The corresponding risk when CMR was accounted for was 9.86% ± 12.93 (CI: 8.71–11.01) using LDL-C indicator and 9.41% ± 12.66 (CI: 8.43–10.39) using Tchol indicator. Using LDL-C or Tchol for either CMR or 10-year risk of CVD did not show any significant difference. 

The 10-year CVD risk was significantly greater using CMR compared to the 10-year risk of CVD based on only FRS (using Tchol indicator). However, the estimate was not significantly greater when LDL-C was used as an indicator. In the current study, the risk of CVD considering different sociodemographic characteristics or dietary intake was reported using CMR with LDL-C indicator.

### 3.2. Sociodemographic Characteristics of 10-Year Risk of CVD in Canadian Adults

The risk of CVD was not significantly different across sex groups (Table 1). CVD risk increased significantly by increase in age and decrease in the levels of education and physical activity (Table 1). Individuals from the highest-income level households were significantly less at risk compared to those from upper-middle income families. In addition, the risk was greater in smokers and White Canadians compared to nonsmokers and non-White Canadians, respectively (*P* < 0.05) (Table 1). 

### 3.3. Dietary Intake and 10-Year Risk of CVD

**Figure 1:**
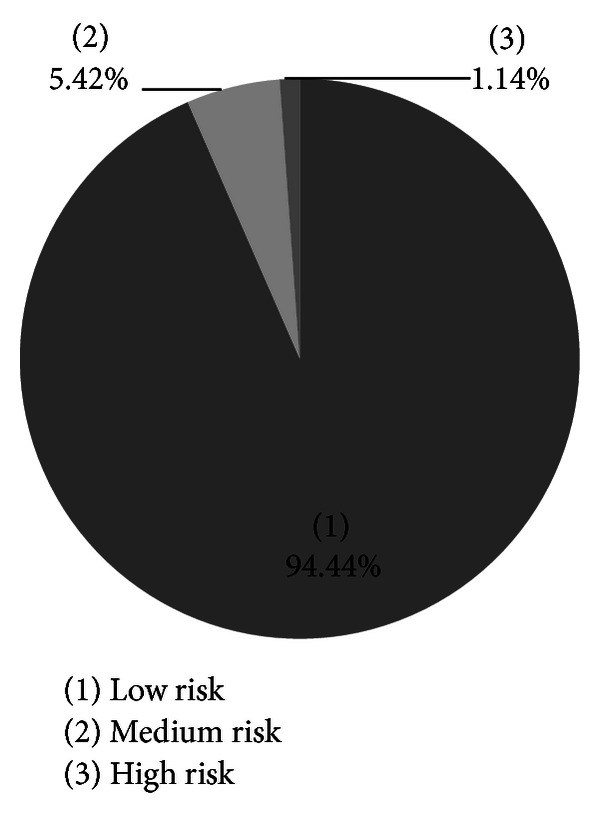
10-year risk of CVD in Canadian adults, 30–74 y, Canadian Health Measures Survey (CHMS), cycle 1, 2007–2009.

Most Canadian adults were in the low CVD risk level (93.4%) (Figure 1). The only significant association was observed in fruit and vegetable juice intake; Canadians at medium risk of CVD consumed less amounts of fruit and vegetable juice compared to their counterparts at low risk (Table 2). 

## 4. Discussion

Overall the 10-year risk of CVD in Canadian adults accounted for 9.86% considering individuals identified with MetS. The risk of CVD increased with age or by a decrease in the level of education (Table 1). The CVD risk was greater among upper-middle income households compared to higher-income households. Additionally, active Canadians with EE ≥ 3 had significantly lower risk compared to their inactive counterparts with 0 ≤ EE < 1.5. Canadians with medium risk of CVD significantly consumed less fruit and vegetable juice compared to Canadians with low risk of CVD.

The introduction to the concept of cardiometabolic risk (CMR) and its risk factors which are not included in traditional risk assessment tools such as FRS, as well as the guidelines for identification and management, was published in a position paper by the Canadian cardiometabolic risk Working Group, in 2009 [6]. The authors believe that traditional risk assessment tools solely underestimated the absolute risk of CVD at an individual level. According to this concept, the absolute CVD risk can be obtained from the algorithms validated by the observational cohort studies (e.g., FRS) taking into account individuals identified with MetS [6]. MetS imparts a relative increase in risk of CVD about 1.5- to 2-fold.

Evaluating the 10-year risk of CVD is usually conducted in prospective studies. Our study and one study among American adults [25] used a cross-sectional design which might be considered as a limitation. The estimate of 9.86% of 10-year CVD risk among Canadian adult population in the current study was similar to American and European prospective studies [26, 27]. Approximately 9.05% of the 12,089 Black and White middle-aged individuals, participated in Atherosclerosis Risk in Communities (ARIC) study after 11 years of follow-up, developed ischemic stroke and coronary heart disease (CHD) [26]. The corresponding prevalence in the European cohorts of the seven-country study, comprised of men aged 40–59 y after 10 years of follow-up, accounted for 9.69% for fatal or nonfatal CVD [27]. Additionally, the earlier study among Americans indicated 11.41% incidence of CHD after 12 years of follow-up through Framingham Heart Study which shows a decrease in the incidence of CVD among Americans over time [28]. Therefore, since the reports from prospective studies were similar to our finding, it might be possible to use the estimate of 10-year risk of CVD through cross-sectional studies which are more feasible than prospective studies. 

Our finding suggests including CMR in assessing the risk of CVD not only at individual level but also at population level. The similar estimates using Tchol and LDL-C indicate the importance of simple measure of Tchol in this assessment at population level. Assessing CMR by using a simple measure of Tchol allows avoiding the underestimation of CVD risk in individuals with MetS. Our study showed only a small overlap in the 95% confidence interval of the two estimates when LDL-C was used, which likely relates to the small sample size. 

Older age was considered as one of the strong factors in elevating the risk of CVD, as observed in our study. The greater CVD risk in older adults is not surprising as this group usually has higher prevalence of most CVD defining components compared to other adults. A similar finding was seen among American adults 20 years and older who participated in a cross-sectional sample of 5,440 in the National Health and Nutrition Examination Surveys (NHANES) 1999–2004 [29]. According to NHANES the prevalence of metabolic abnormalities increased with age among all individuals. In this study they used elevated blood pressure, elevated triglyceride and glucose levels, insulin resistance, systemic inflammation, and decreased HDL-C level as cardiometabolic abnormalities which were comparable to our study. 

Physical activity in the current study was based on the energy expenditure during leisure time. Specifically, the significant difference in CVD risk between active and inactive individuals was similar to the finding of a Multiple Risk Factor Intervention Trial [30]. Among 12,138 middle-aged men measuring self-selected leisure-time physical activity (LTPA), combined fatal and nonfatal major CHD events were 20% lower with high activity as compared with low LTPA [30]. Moreover, better cardiometabolic characteristics such as lower heart rate, lower body mass index, and a higher HDL-C level or lower risk of CHD have been observed in more active women from two cohort studies than less active ones [31, 32]. Although Lee et al. 2001 [32] indicated that vigorous intensity of physical activity had a significant impact on reducing the risk of CHD compared to the lowest intensity, regular walking independently predicted lower risk [32]. The significant role of physical activity is more evident when the impact has still remained after adjustment for potential confounders such as smoking status, diet, and alcohol use in most studies. Physical activity independently, regardless of obesity, could contribute to the development of CHD in women [33]. 

In our study, risk of CVD was about 1.5 times greater in smokers than nonsmokers. In general, there is a nonlinear relation between the risk of CVD and the number of cigarettes smoked daily [34, 35]. The mechanisms that cause acute cardiovascular events in smokers include increased hypercoagulability leading to thrombosis, endothelial dysfunction, and development of chronic inflammatory state by an increase in white cells and CRP values [36]. In studies evaluating the impact of physical activity and risk of CVD, the interaction between physical activity and smoking has been reported [31, 32]. Among American women from Women's Health Study, physical activity was inversely associated with CHD rate in current and past smokers but not in nonsmokers [32]. Additionally, subjects from Framingham Offspring Study who were more active tended to smoke fewer cigarettes [31]. On the other hand, the impact of physical activity and smoking on the risk of CVD cannot be reported hierarchically; that is, each has its own strong impact. 

Others report that current rather than childhood socioeconomic status has more influence on most cardiometabolic risk factors: physical inactivity, HDL-C, triglycerides, post-load glucose, fibrinogen, and smoking [37]. Individuals in households with high level of education and income had lower risk of CVD in the current study. Education had a greater impact on the risk of CVD; that is, a decreasing trend in the CVD risk was observed by raising the level of education. The greater impact of education might be explained by its unchangeable virtue (occupation or income to). Thus, education is the most frequent measure used to evaluate the impact of socioeconomic status and cardiometabolic abnormalities [38]. According to a review by Kaplan and Keil, 1993, during 40 years of study there has been a consistent inverse relation between cardiovascular disease, primarily coronary heart disease, and many of the indicators of socioeconomic status [38]. Therefore, it would be necessary to control the impact of socio-economic status while evaluating the association between cardiometabolic risk and potential variables.

The dietary intake was compared only between individuals with medium risk of CVD and individuals with low risk of CVD, due to the small number categorized as high risk. The only significant difference in the dietary consumption was found in fruit and vegetable juice intake, with medium risk group having a decreased intake of fruit and vegetable juice. This finding is likely due to the adherence of people with diagnosed disease such as diabetes to a special diet [39]. In our study on MetS among Canadian population using CHMS data, we found that diabetics significantly consumed less fruit and vegetable juice compared to individuals with no diagnosis of diabetes [13]. Diabetics are reported to pay more attention to food labels, especially to the sugar information, than those without diabetes [39]. Diabetics in the Multiethnic Cohort Study (MEC) had lower consumption of juice (>10% difference) compared to those without diabetes [40]. The Canadian Diabetes Association recommends that diabetics: “have vegetables and fruit more often than juice” [41].

*Limitations*. The approach of determining the absolute risk considering relative factor of about 1.5 to 2 in individuals with MetS is not fully validated in longitudinal studies, rather it is based on studies reporting that the presence of the metabolic syndrome is associated with a 1.5–2 increased risk, above and beyond Framingham Risk Score. According to Guize et al., 2007, the prevalence of MetS or the risk of all-cause mortality is different using different combinations of MetS diagnostic criteria [42]. However, this finding needs further investigation considering different factors such as ethnicity, lifestyle factors, and underlying disease. To be able to include all indicators, we had to use the fasted CHMS subsample. This along with the exclusion criteria made the sample size of the study smaller than the whole CHMS sample. Further cycles of CHMS in upcoming years are needed to compare the dietary intake between three levels of CVD risk with a greater sample size, especially in high risk group. Although CHMS collected usual dietary intake through semiquantitative questionnaires, the quantity of the consumption is not provided to obtain an exact measure of dietary intake. To generalize data to the Canadian population, we used the specific weights provided in CHMS for fasted subsample. Moreover, since CHMS has provided fasted subsample, we were able to compare the prevalence of 10-year risk of CVD using either LDL-C (present in fasted subsample) or Tchol (present in both fasted and nonfasted subsample). 

## 5. Conclusion

The 10-year risk of CVD in Canadian adults population aged from 30 to 74 years significantly changed when we considered CMR (8.10% versus 9.86%, resp.). In fact, the absolute CVD risk obtained from the validated traditional risk assessment tools might underestimate the 10-year risk of CVD in Canadian adult population. Compared to prospective studies, using cross-sectional study may also give similar estimation of the 10-year risk of CVD. The 10-year risk of CVD was greater in smokers, individuals with low physical activity, or with low level of education. These factors need to be controlled when evaluating the association between cardiometabolic risk and potential factors. Dietary intake across three levels of 10-year risk of CVD showed a significant difference in fruit and vegetable juice intake between low and medium risk of CVD, with individuals at medium risk having less intake. However, larger studies are needed to evaluate the difference in dietary patterns among individuals at different levels of CVD risk. 

## Figures and Tables

**Table 1:** Weighted estimates of 10-year risk of CVD or cardiometabolic risk (CMR) by sociodemographic characteristics of Canadians aged from 30 to 74 years Canadian Health Measures Survey, Cycle 1, 2007–2009 (*n* = 1,293).

Characteristics	10-year risk of CVD, percent (SE^†^)	Confidence intervals (CIs)
Sex		
Male	8.72 (0.43)	7.77–9.67
Female	10.92 (1.07)	8.56–13.28
Age^∗∗1^		
(1) 30–34 years	1.95 (0.10)	1.72–2.17
(2) 35–39 years	2.95 (0.40)	2.07–3.84
(3) 40–44 years	5.42 (0.59)	4.11–6.73
(4) 45–49 years	6.02 (0.39)	5.15–6.88
(5) 50–54 years	11.91 (2.21)	7.03–16.79
(6) 55–59 years	14.32 (1.80)	10.34–18.30
(7) 60–64 years	19.83 (2.07)	15.26–24.40
(8) 65–69 years	20.68 (0.94)	18.60–22.75
(9) 70–74 years	24.68 (2.93)	18.21–31.14
Education level^∗∗2^		
Less than secondary school graduation	19.35 (3.69)	11.20–27.49
Secondary school graduation	14.85 (1.52)	11.49–18.20
Some postsecondary	8.76 (0.78)	7.04–10.49
Postsecondary graduation	8.50 (0.44)	7.52–9.47
Income level^∗∗3^		
Lowest income	11.10 (3.04)	4.39–17.81
Lower-middle income	12.06 (1.72)	8.26–15.86
Upper-middle income	11.34 (0.81)	9.53–13.14
Highest income	8.10 (0.49)	7.01–9.20
Physical activity^∗∗4^		
Inactive	10.80 (0.78)	9.06–12.54
Moderately active	9.66 (0.66)	8.19–11.13
Active	7.46 (0.52)	6.30–8.63
Alcohol		
Never drink	10.74 (2.42)	5.41–16.08
Ever drink	9.81 (0.48)	8.74–10.87
Ethnicity*		
Non-White	7.32 (0.89)	5.35–9.30
White	10.32 (0.53)	9.15–11.49
Smoking*		
Nonsmokers	8.87 (0.32)	8.15–9.60
Smokers	13.18 (1.52)	9.83–16.53

^†^SE: standard error.

**Significant (*P* < 0.05), 95% confidence interval overlap.

*Significant (*P* < 0.05), independent *t*-test.

^1^According to 95% confidence interval, the risk of CVD in age groups 2 and 3 was significantly different. Age groups 5, 6, 7, 8, and 9 were significantly different from age groups 1, 2, 3, and 4. In addition, age group 9 and 8 were significantly different from age group 5.

^2^According to 95% confidence interval, the risk of CVD was significantly less among Canadians with some postsecondary education or postsecondary graduation compared to Canadians with less than secondary school graduation or secondary school graduation.

^3^The risk of CVD was significantly less among Canadians with highest income compared to upper-middle income.

**Table 2:** Dietary consumption among Canadians aged from 30 to 74 years at different level of 10-year risk of CVD, Canadian Health Measures Survey, Cycle 1, 2007–2009 (*n* = 1,293).

Food and beverages times/day^1^	Low 10-year CVD risk (*n* = 1190)	Medium 10-year CVD risk (*n* = 84)	High 10-year CVD risk (*n* = 19)
		Mean of intake (SE^2^) CIs^3^	
Meat and fish			
Red meat, organs, hotdogs, sausage or bacon, seas foods, eggs, beans, and nuts	1.81 (0.13)	1.31 (0.08)	1.95 (0.42)
	1.50–2.11	1.12–1.49	1.01–2.89
Grains, fruit, and vegetable			
Hot/cold cereal, white bread, brown bread, rice, and pasta (grains)	2.87 (0.13)	3.76 (1.24)	3.35 (1.84)
	2.56–3.18	1.02–6.50	−0.71–7.41
Fruit and vegetable	3.78 (0.08)	3.65 (0.18)	3.95 (0.45)
	3.59–3.98	3.25–4.05	2.94–4.96
Milk and dairy products			
Milk, cottage cheese, and yoghurt or ice cream	1.52 (0.05)	1.59 (0.18)	1.44 (0.22)
	1.41–1.63	1.17–2.00	0.94–1.95
Dietary fat			
Regular-fat salad dressing or mayonnaise and regular-fat potato chips, tortilla chips, or corn chips	0.42 (0.02)	0.37 (0.07)	0.37 (0.08)
	0.38–0.47	0.21–0.53	0.18–0.55
Water and soft drinks			
Regular soft drink, sport drink, and fruit drink (sugar-sweetened beverages)	0.39 (0.05)	0.22 (0.05)	0.15 (0.06)
	0.28–0.50	0.11–0.34	0.01–0.29
Diet soft drink	0.16 (0.01)	0.31 (0.11)	0.39 (0.17)
	0.12–0.20	0.06–0.55	0.01–0.77
Fruit and vegetable juice*	0.71 (0.03)	0.43 (0.09)	1.46 (0.78)
	0.64–0.79	0.23–0.63	−0.26–3.19

^1^Frequency of consumption.

^2^SE: standard error.

^3^Confidence intervals.

*Significant (*P* < 0.05), based on 95% confidence interval between low risk versus medium risk.

## References

[1] Public Health Agency of Canada http://www.phac-aspc.gc.ca/cd-mc/cvd-mcv/cvd-mcv-eng.php.

[2] Heart and Stroke Foundation http://www.heartandstroke.com/site/c.ikIQLcMWJtE/b.3483991/k.34A8/Statistics.htm.

[3] Sheridan S, Pignone M, Mulrow C (2003). Framingham-based tools to calculate the global risk of coronary heart disease. *Journal of General Internal Medicine*.

[4] Wilson PWF, D'Agostino RB, Levy D, Belanger AM, Silbershatz H, Kannel WB (1998). Prediction of coronary heart disease using risk factor categories. *Circulation*.

[5] Leiter LA, Fitchett DH, Gupta REG (2011). Identification and management of cardiometabolic risk in Canada: a position paper by the cardiometabolic risk working group. *The Canadian Journal of Cardiology*.

[6] Leiter LA, Fitchett DH, Gilbert RE (2011). Cardiometabolic risk in Canada: a detailed analysis and position paper by the cardiometabolic risk working group. *The Canadian journal of cardiology*.

[7] Grundy SM (2008). Metabolic syndrome pandemic. *Arteriosclerosis, Thrombosis, and Vascular Biology*.

[8] Brien SE, Katzmarzyk PT (2006). Physical activity and the metabolic syndrome in Canada. *Applied Physiology, Nutrition and Metabolism*.

[9] Galassi A, Reynolds K, He J (2006). Metabolic syndrome and risk of cardiovascular disease: a meta-analysis. *The American Journal of Medicine*.

[10] Gami AS, Witt BJ, Howard DE (2007). Metabolic syndrome and risk of incident cardiovascular events and death: a systematic review and meta-analysis of longitudinal studies. *Journal of the American College of Cardiology*.

[11] Dekker JM, Girman C, Rhodes T (2005). Metabolic syndrome and 10-year cardiovascular disease risk in the Hoorn study. *Circulation*.

[12] Stern MP, Williams K, González-Villalpando C, Hunt KJ, Haffner SM (2004). Does the metabolic-syndrome improve identification of individuals at risk of type 2 diabetes and/or cardiovascular disease?. *Diabetes Care*.

[13] Setayeshgar S, Whiting SJ, Vatanparast H (2012). Metabolic syndrome in Canadian adults and adolescents: prevalence and associated dietary intake. *International Scholarly Research Network Obesity*.

[14] Obarzanek E, Sacks FM, Vollmer WM (2001). Effects on blood lipids of a blood pressure-lowering diet: the dietary approaches to stop hypertension (DASH) trial. *American Journal of Clinical Nutrition*.

[15] Sacks FM, Svetkey LP, Vollmer WM (2001). Effects on blood pressure of reduced dietary sodium and the dietary approaches to stop hypertension (dash) diet. *The New England Journal of Medicine*.

[16] Panagiotakos DB, Pitsavos C, Stefanadis C (2006). Dietary patterns: a mediterranean diet score and its relation to clinical and biological markers of cardiovascular disease risk. *Nutrition, Metabolism and Cardiovascular Diseases*.

[17] Mente A, De Koning L, Shannon HS, Anand SS (2009). A systematic review of the evidence supporting a causal link between dietary factors and coronary heart disease. *Archives of Internal Medicine*.

[18] Giroux S (2007). Canadian health measures survey: sampling strategy overview. *Health Report*.

[19] Wilson PW, D'Agostino RB, Levy D D, Belanger AM, Silbershatz H, Kannel WB (1998). Prediction of coronary heart disease using risk factor categories. *Circulation*.

[20] Nathan DM (2009). International expert committee report on the role of the A1C assay in the diagnosis of diabetes: response to kilpatrick, bloomgarden, and zimmet. *Diabetes Care*.

[21] Alberti KG, Eckel RH, Grundy SM (2009). Harmonizing the metabolic syndrome. *Circulation*.

[22] Canadian Health measures survey Cycle 1, 2007–2009, household questionnaire. http://www23.statcan.gc.ca/imdb-bmdi/pub/instrument/5071_Q1_V1-eng.pdf.

[23] National Population Health Survey http://www23.statcan.gc.ca/imdb/p2SV.pl?Function=getSurvey&SDDS=3225&lang=en&db=imdb&adm=8&dis=2.

[24] Schenker N, Gentleman JF (2001). On judging the significance of differences by examining the overlap between confidence intervals. *The American Statistician*.

[25] Ford ES, Wayne MS, Giles WH, Mokdad AH (2004). The distribution of 10-year risk for coronary heart disease among U.S. adults. *Journal of the American College of Cardiology*.

[26] McNeill AM, Rosamond WD, Girman CJ (2005). The metabolic syndrome and 11-year risk of incident cardiovascular disease in the atherosclerosis risk in communities study. *Diabetes Care*.

[27] Menotti A, Lanti M, Puddu PE, Kromhout D (2000). Coronary heart disease incidence in northern and southern European populations: a reanalysis of the seven countries study for a European coronary risk chart. *Heart*.

[28] Wilson PWF, D’Agostino RB, Levy D, Belanger AM, Silbershatz H, Kannel WB (1998). Prediction of coronary heart disease using risk factor categories. *Circulation*.

[29] Wildman RP, Muntner P, Reynolds K (2008). The obese without cardiometabolic risk factor clustering and the normal weight with cardiometabolic risk factor clustering: Prevalence and correlates of 2 phenotypes among the US population (NHANES 1999–2004). *Archives of Internal Medicine*.

[30] Leon AS, Connett J, Jacobs DR, Rauramaa R (1987). Leisure-time physical activity levels and risk of coronary heart disease and death, the multiple risk factor intervention trial. *Journal of the American Medical Association*.

[31] Dannenberg AL, Keller JB, Wilson PWF, Castelli WP (1989). Leisure time physical activity in the Framingham Offspring study description, seasonal variation, and risk factor correlates. *American Journal of Epidemiology*.

[32] Lee IM, Rexrode KM, Cook NR, Manson JE, Buring JE (2001). Physical activity and coronary heart disease in women: is “No Pain, No Gain” passé?. *Journal of the American Medical Association*.

[33] Li TY, Rana JS, Manson JE (2006). Obesity as compared with physical activity in predicting risk of coronary heart disease in women. *Circulation*.

[34] Burns DM (2003). Epidemiology of smoking-induced cardiovascular disease. *Progress in Cardiovascular Diseases*.

[35] Willett WC, Green A, Stampfer MJ (1987). Relative and absolute excess risks of coronary heart disease among women who smoke cigarettes. *The New England Journal of Medicine*.

[36] Pyrgakis VN (2009). Smoking and cardiovascular disease. *Hellenic Journal of Cardiology*.

[37] Brunner E, Shipley MJ, Blane D, Davey Smith G, Marmot MG (1999). When does cardiovascular risk start? Past and present socioeconomic circumstances and risk factors in adulthood. *Journal of Epidemiology and Community Health*.

[38] Kaplan GA, Keil JE (1993). Socioeconomic factors and cardiovascular disease: a review of the literature. *Circulation*.

[39] Fitzgerald N, Damio G, Segura-Pérez S, Pérez-Escamilla R (2008). Nutrition knowledge, food label use, and food intake patterns among latinas with and without type 2 diabetes. *Journal of the American Dietetic Association*.

[40] Nöthlings U, Boeing H, Maskarinec G (2011). Food intake of individuals with and without diabetes across different countries and ethnic groups. *European Journal of Clinical Nutrition*.

[41] Canadian Diabetes Association (2008). 2008 clinical practice guidelines for the prevention and management of diabetes in Canada. *Canadian Journal of Diabetes*.

[42] Guize L, Thomas F, Pannier B, Bean K, Jego B, Benetos A (2007). All-cause mortality associated with specific combinations of the metabolic syndrome according to recent definitions. *Diabetes Care*.

